# Extracellular Vesicles from Mesenchymal Stem Cells: Towards Novel Therapeutic Strategies for Neurodegenerative Diseases

**DOI:** 10.3390/ijms24032917

**Published:** 2023-02-02

**Authors:** Ermanna Turano, Ilaria Scambi, Federica Virla, Bruno Bonetti, Raffaella Mariotti

**Affiliations:** 1 Department of Neuroscience, Biomedicine and Movement Sciences, University of Verona, 37134 Verona, Italy; 2 Neurology Unit, Azienda Ospedaliera Universitaria Integrata Verona, 37124 Verona, Italy

**Keywords:** mesenchymal stem cells, extracellular vesicles, exosomes, neurodegenerative disease, intranasal administration

## Abstract

Neurodegenerative diseases are fatal disorders of the central nervous system (CNS) which currently lack effective treatments. The application of mesenchymal stem cells (MSCs) represents a new promising approach for treating these incurable disorders. Growing evidence suggest that the therapeutic effects of MSCs are due to the secretion of neurotrophic molecules through extracellular vesicles. The extracellular vesicles produced by MSCs (MSC-EVs) have valuable innate properties deriving from parental cells and could be exploited as cell-free treatments for many neurological diseases. In particular, thanks to their small size, they are able to overcome biological barriers and reach lesion sites inside the CNS. They have a considerable pharmacokinetic and safety profile, avoiding the critical issues related to the fate of cells following transplantation. This review discusses the therapeutic potential of MSC-EVs in the treatment of neurodegenerative diseases, focusing on the strategies to further enhance their beneficial effects such as tracking methods, bioengineering applications, with particular attention to intranasal delivery as a feasible strategy to deliver MSC-EVs directly to the CNS in an effective and minimally invasive way. Current progresses and limiting issues to the extent of the use of MSC-EVs treatment for human neurodegenerative diseases will be also revised.

## 1. Introduction

The treatment of most neurological disorders currently represents a therapeutic challenge for the researchers committed to improving and/or extending the quality and lifespan of affected people.

Neurodegenerative diseases such as Alzheimer’s disease (AD), Parkinson’s disease (PD), and amyotrophic lateral sclerosis (ALS) are characterized by the accumulation of specific proteins within the nervous system accompanied by a progressive loss of neurons in the affected regions [1,2]. The pathogenic mechanisms are still unclear and the failure to identify the precise causes of neuronal degeneration leads to the absence of treatments [3].

The use of stem cells was considered a potentially suitable strategy for the treatment of neurodegenerative disorders. Stem cells are undifferentiated totipotent or multipotent cells that can be obtained from a variety of adult tissues such as bone marrow, brain, liver, skin, skeletal muscle, gastrointestinal tract, pancreas, eye, blood, fat, and dental pulp [4]. The mesenchymal stem cells (MSCs) can differentiate to osteoblasts, adipocytes, and chondrocytes in vitro [5]. Due to their capacity to transdifferentiate in vitro into epithelial cells and lineages derived from the neuroectoderm, MSCs have been considered to be able to repair injured, damaged, or diseased tissues [6]. Moreover, MSCs possess the important ability to modulate the immune response of a broad range of immune cells, both in vitro and in vivo [7,8,9,10]. The use of MSCs for tissue repair requires such cells to be able to easily access the target organ. Several works have demonstrated their ability to home into the damaged brain, migrating from the blood toward inflamed tissues where they exert a neuroprotective effect [11,12,13].

The efficacy of MSCs in neurological diseases was demonstrated in several preclinical studies [14,15]. However, despite their therapeutic action, the engraftment of MSCs in central nervous system (CNS) tissues, after their transplantation, results in a small percentage of differentiated, detectable MSCs [16,17]. These considerations suggest that the ability of MSCs to modify the tissue microenvironment via secretion of soluble factors may contribute more significantly to tissue repair than their capacity of transdifferentiation [18,19,20,21].

**Figure 1:**
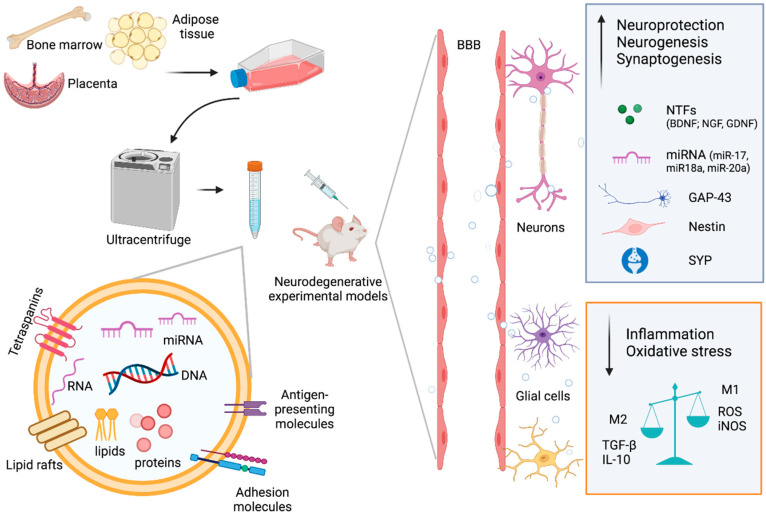
Extracellular vesicles from mesenchymal stem cells as an innovative therapy for neurodegenerative disease. MSC-EVs derived from different cellular sources, due to their small size, cross the blood–brain barrier and reach the affected cells of the diseased brain. Here, EVs release neurotrophic factors (NTFs), miRNA, and anti-inflammatory molecules that mediate neuroprotection, neurogenesis, synaptogenesis, and decrease the neuroinflammation. EVs with their cargo contribute to a functional recovery and neurodegeneration reduction. BBB = blood–brain barrier; NTFs = neurotrophic factors; TGF-β = transforming growth factor-β; IL-10 = interleukin-10; GAP-43 = growth-associated protein-43; SYP = synaptophysin; ROS = reactive oxygen species; iNOS = inducible nitric oxide synthase; M1 = pro-inflammatory M1-polarized microglia; M2 = anti-inflammatory M2-polarized microglia.

Such action is achieved through a paracrine mechanism, the release of extracellular vesicles (EVs), which is, indeed, common to almost all cells. EVs are membranous structures derived from the endosomal system or shedding from the plasma membrane [22,23,24,25] whose release and uptake provide a novel mechanism of transcellular communication [26,27]. MSCs are also able to release a large number of EVs with high therapeutic power which constitute an effective alternative to cell-therapy in neurodegenerative diseases, due to their content which can reproduce the effect of their parental cells [28,29,30] (Figure 1). Indeed, MSC-EVs contain many neurotrophic factors (NTFs), immunomodulatory and anti-inflammatory molecules including transforming growth factor-β (TGF-β), and interleukin-10 (IL-10), which are involved in favoring the processes of neurogenesis and neuroprotection and promote functional recovery [31,32,33]. Interestingly, proteins involved in neural development and synaptogenesis, such as nestin, growth-associated protein 43 (GAP-43), and synaptophysin are incorporated in MSC-EVs [34]. Moreover, in terms of miRNA content, a specific signature of miRNAs was reported, which was implicated in promoting CNS recovery by modulating neurogenesis and stimulating axonal growth [35].

In 2018, the International Society for Extracellular vesicles (ISEV) updated the guidelines for the study of EVs. They are a heterogeneous population whose size may vary typically between 50 nm and 500 nm, but they can be even larger, measuring 1–10 μm. The ISEV recommends the use of appropriate nomenclature for EVs, classifying them by clear, measurable characteristics (such as cell of origin, molecular markers, size, etc.) thus abandoning terms such as “exosomes” or “microvesicles” that were previously used [23,36,37].

EVs are present in many biological fluids, including blood, CSF, urine, saliva, and amniotic fluid, as well as in the conditioned medium of cell culture [38,39]. Their role was originally thought to be a source of cellular dumping; however, it has since been found that EVs play important roles in participating in cell-to-cell communication via the transfer of membrane receptors, proteins, lipids, and RNAs between cells and also in cell maintenance and tumor progression [40,41]. The function of small EVs depends on their ability to interact with recipient cells and to deliver their contents to such cells [42].

Thanks to their small size, which allows them to pass the blood–brain barrier (BBB) and deliver their cargo (biological or pharmacological) to the brain, they become a powerful therapeutic application tool in neurodegenerative diseases where the BBB represents the main obstacle to reach the injured area of CNS.

In this review, we summarize encompassing information about the therapeutic properties of EVs from MSCs to treat debilitating and fatal neurodegenerative disorders.

## 2. EVs from MSCs

It has been shown that MSC secreted factors are able not only to improve the surrounding environment of the target tissue, but also to exert beneficial effects even in the distal sites, supporting the paracrine hypothesis rather than the local transdifferentiation one [43,44]. The paracrine effects of MSCs were first described in 2006 and took into account the entire secretome released by MSCs, which contains a soluble fraction (mostly growth factors and cytokines) and a vesicular component, EVs, which transfer proteins, lipids, and genetic material to recipient cells [45,46].

MSCs are considered prolific producers of EVs compared to other types of cells [47,48] and the therapeutic use of their vesicular counterpart shows significant advantages over using parental cells, thanks to a high safety profile, low immunogenicity, and tumorgenicity [49]. The composition of MSC-derived EVs, like EVs from other sources, includes a cargo of nucleic acids, proteins, and lipids reported in several studies and in databases such as VESICLIPEDIA (http://microvesicles.org/) [50] or EXOCARTA (www.exocarta.org) [51] both accessed on 25 January 2023, a curated compendium of molecular data. The phenotype, number, and function of MSC-EVs may vary depending on the source of MSCs [52,53]. Thanks to their small size, MSC-EVs are able to migrate efficiently to the target organ after infusion, without getting trapped in pulmonary capillaries [54], crossing the BBB, and reaching the injured area in the brain. These characteristics make MSC-EVs a promising tool for a cell-free therapy in neurodegenerative diseases.

## 3. Therapeutic Potential of MSC-EVs in Neurodegenerative Disorders

Neurodegenerative diseases are a heterogeneous group of disorders that affect approximately 30 million individuals worldwide with distinct morphological and pathophysiological features. These diseases have a complex multifactorial etiology whose pathogenic mechanisms are currently not fully understood [55,56]. The pathological conditions arise from slow progressive and irreversible dysfunctions caused by loss of both neurons and synapses in selected areas of the nervous system. A combination of genetic and environmental factors may play a role in causing neurodegenerative diseases [57]. The incidence of neurological disorders becomes more widespread with the aging of the population [58] and results to be closely related to lifestyle factors. Likewise, environmental factors are recognized among the causes of disease and progression, and include chronic exposure to heavy metals, pesticides, and air pollutants [59,60].

Although neurodegenerative diseases present distinct characteristics, common pathways have been identified, through which the neurodegeneration proceeds. Common pathogenic mechanisms underlying many neurodegenerative diseases include abnormal accumulation of insoluble protein aggregates and misfolding [61], oxidative stress and formation of reactive oxygen species (ROS) [62], mitochondrial dysfunctions [63], and neuroinflammatory processes [64], suggesting that neurodegenerative diseases with distinct etiologies may share common pathogenic pathways [65].

Currently, no neurodegenerative disease is curable, and the available treatments only manage the symptoms or delay the progression of the disease [3].

A large number of neurodegenerative disorders are characterized neuropathologically by intracellular and/or extracellular aggregates of proteinaceous fibrils which are implicated in progressive brain degeneration [66]. For instance, the accumulation of amyloid beta (Aβ) together with the presence of neurofibrillary tangles, synapses, and neuronal loss, correlates with a progressive and gradual decline in cognitive functions, typical of Alzheimer disease (AD) [67]. Several therapeutic approaches have attempted to reduce the Aβ burden in AD patients and in transgenic mouse models: the presence of high levels of Aβ-degrading enzymes in adipose MSC-derived EVs has been considered a useful strategy to regulate the level of Aβ accumulation in the brain [68]. Indeed, EVs isolated from human umbilical cord-derived MSCs significantly enhance the expression of Aβ-degrading enzymes such as neprilysin (NEP) and insulin degrading enzyme (IEP), reducing Aβ deposition of AD in transgenic APP/PS1 mice, with a subsequent reduction in neuroinflammation and cognitive improvement [69]. Moreover, the content of antioxidant enzymes, such as the catalase in MSC-EVs, protects hippocampal neurons from oxidative stress and synaptic damage [70].

The use of MSC-EVs has been reported to act against neuronal damage and synaptic dysfunction [71], pathological signs that generally appear in the initial phase of AD, which are directly related to cognitive impairment. In this context, MSC-EVs have been shown to promote neuroprotection [72] and stimulate neurogenesis [73].

A similar scenario is envisaged with Parkinson’s disease (PD), the second most common chronic neurodegenerative disease in the world [74], characterized by the degeneration of dopaminergic neurons with the accumulation of protein aggregates of α-synuclein in the intraneuronal structure, and a consequent deficiency of dopamine production in several networks [75]. The use of MSC-EVs as a therapeutic strategy turns out to be promising, although still at an early stage. The use of the secretome from MSCs showed, in PD rat models, an improvement in motor performance outcomes [76,77]. MSC-EVs from different sources were able to promote neuroprotection of 6-hydroxy-dopamine (6-OHDA) dopaminergic neurons from oxidative stress [78], reducing substantia nigra dopaminergic neuron loss, apoptosis, and upregulating the dopamine levels in the striatum [79].

The considerable capabilities of MSC-EVs have been observed in other settings: EVs derived from adipose mesenchymal stem cells (ASCs) showed the ability to promote remyelination after injury and neuroprotection of neurons and motor neuron-like cells, after peroxide treatment in vitro [80,81], demonstrating their potential therapeutic application in several neurodegenerative diseases. In particular, motoneurons (MNs) represent the principal target of amyotrophic lateral sclerosis (ALS), due to the selective dysfunction and damage of upper and lower MNs leading to progressive paralysis and death [82]. ASC-EVs have demonstrated the ability to regulate the aggregation of the pathological SOD1 protein restoring the levels of mitochondrial proteins in neurons from G93A mutated ALS mice [83] and in MN cultures, an effect that is due to their antiapoptotic ability [84]. MN and neuromuscular junction protection, together with an improvement in motor performance was observed in SOD1(G93A) mice after repeated administration of ASC-EVs [85].

As previously reported, the regulation of ROS production plays an important role among the pathogenic mechanisms of neurodegenerative diseases. With regard to this, recently, the role of MSC-EVs in reducing oxidative and nitrosative damage has drawn much attention. Antioxidant effects have been observed in models of PD [86] and in alcohol-related brain damage [87], having effects on several cell types including neurons and glial cells [86], as well as effects reported on brain ischemic injury [88]. This evidence suggests a potential mechanism of action of MSC-EVs to counteract ROS-related damage that causes neurodegeneration. The ability to act against mitochondrial dysfunction is also reported to be a mechanism of action to counteract neurodegeneration [84,89], as well as the capacity of MSC-EVs to counteract the accumulation of aberrant proteins as previously described for Aβ accumulation in AD and α-synuclein aggregation in PD. Furthermore, MSC-EVs are able to act as modulators of the inflammatory component, whose role in the progression of neurodegenerative diseases is currently being re-evaluated.

Inflammation associated with chronic neurodegenerative diseases is not typically the trigger itself of such diseases; however, it contributes and sustains their progress due to the contribution of activated microglia and astrocytes in neuronal dysfunction and death [64]. MSC-EVs elicited a strong anti-inflammatory effect in an AD mouse model, improving the amount of M2-polarized macrophages. A reduction in inducible nitric oxide synthase (iNOS) was observed in ALS mice after MSC-EVs injection [71]. For a more extensive and complete discussion of the mechanisms of action of MSC-EVs refer to Yari et al. [33].

In light of this promising evidence, the use of MSC-EVs in the treatment of neurodegenerative diseases currently appears to be a possible innovative strategy for the treatment of incurable diseases.

## 4. Strategies to Improve the Therapeutic Effect of MSC-EVs

Delivering therapeutic agents efficiently within the CNS represents one of the crucial issues of the therapies for neurodegenerative disorders. The passage through the BBB represents one of the limiting factors in conveying an efficient concentration of therapy in the areas of lesion in the CNS. The latter concern, related to cellular therapies, is strictly linked to the homing and biodistribution of transplanted cells and their vesicular counterpart, as well as to the identification of an efficient method of administration (Table 1).

### 4.1. MSC-EVs Labeling and Tracking Methods

MSC-EVs have recently been shown to have intrinsic homing capabilities similar to their parental cells [111] and, due to their very small size, they are able to bypass the BBB [112,113].

Scientists devoted considerable efforts to identify methods for the labeling and detection of EVs when injected systemically: the use of fluorescent dyes [90,91,114], as well as the use of magnetic imaging techniques and tomography [93,96,97,98] represent appealing methods to detect EVs in vivo. In particular, MRI, widely used for clinic purposes, does not require the use of ionizing radiation while maintaining a good soft tissue contrast. In order to be visualized with molecular imaging methods such as MRI, EVs must be loaded with contrast agents to produce a detectable change in signal intensity [115]. An innovative approach used for labeling MSC-EVs involves the use of ultra-small superparamagnetic iron oxide nanoparticles (USPIOs). This approach preserves their morphology and physiological characteristics [94]. It has been shown that EVs derived from ASCs, conjugated with USPIOs, reached the CNS and accumulated in the typical lesioned brainstem motor nuclei when injected in SOD1(G93A) mice [85]. Recently, Han and co-workers have improved the sensitivity of detection of EVs conjugated with USPIOs, using a platform technology to prepare highly purified magnetically labelled EVs (magneto-EVs). Magneto-EVs can be visualized by MRI following their systemic administration [95].

The identification of EVs is fundamental to evaluate their biodistribution in the target organs, which can be predictive of therapeutic responses. Perets and co-workers recently developed a system to track the migration and homing of intranasally administered small EVs derived from bone marrow MSCs in vivo in different brain diseases. The approach combines classical X-ray computed tomography, with gold nanoparticles as labeling agents. They found that MSC-EVs specifically accumulated in pathologically relevant brain regions of murine models (including stroke, autism, PD, and AD), while in healthy controls, they showed a diffuse migration pattern with rapid clearance. Moreover, the accumulation of MSC-EVs correlates with inflammatory signals in pathological brains [98]. The homing capability of MSC-EVs towards inflammatory signals is strictly related to the markers expressed on their surface, such as integrins. Integrins expressed on EV surfaces have been able to drive vesicles toward specific tissues, as was originally observed in cancer vesicles [116]. In this regard, the use of EV labeling systems which do not perturb the membrane’s integrity, protecting proteins and receptors that are useful for inflammatory chemotaxis, is therefore of main importance. Indeed, it has been shown that treatment with proteinase K on EVs from MSCs impairs the membrane proteins, causing a reduction in the homing of MSC-EVs toward inflamed sites of injury [92]. Similarly, the use of pertussis toxin induces a block of chemokine receptors with subsequent impaired capacity of MSC-EVs to migrate [117]. Using innovative tracking methods and increasing the knowledge of the biological mechanisms underlying the homing of MSC-EVs, it is possible to enhance their therapeutic action through their passage across the BBB, using different delivery routes and applying bioengineering modifications.

### 4.2. Passage through Blood–Brain Barrier

The BBB is a selectively permeable membrane regulating the passage of substances between the peripheral vascular circulation and the CNS, thus serving to protect the CNS from harmful substances or overzealous immune responses [118].

The BBB consists of a monolayer of endothelial cells surrounded by capillaries. Endothelial cells interact with and are bound to the basement membrane, in association with astrocytes and pericytes [119,120]. Endothelial cells and their tight junctions are relatively impermeable, blocking the diffusion of a proportion of large molecules to the CNS.

However, this cerebral compartmentalization represents the major obstacle in the administration of drugs or other therapeutic substances directed towards injured sites in the CNS. Several strategies are being developed to enhance the quantity and concentration of therapeutic agents delivered to the CNS. Gabathuler describes the “physiological approach” as the most efficient one for obtaining a regular distribution of molecules in the brain through the endothelial cells of the BBB. This approach exploits transcytosis due to the presence of specific receptors expressed on the BBB [121]. EVs from different sources, due to their small dimensions, are able to pass the BBB as demonstrated by their capacity to deliver anticancer drugs in neurons [122], and specifically target neural players such as microglia and oligodendrocytes [123].

A recent study showed the therapeutic effect of EVs derived from human neural stem cells in an in vitro model of BBB breakdown. Indeed, 5XFAD AD mice present leakage of the BBB, and EVs were shown to be able to initiate and repair the leakage of the BBB, reversing the deficiency of BBB induced by AD [124].

Nevertheless, relatively few researches identify the precise mechanism of the passage of EVs through the BBB. Some evidence has reported that EVs can cross the BBB using a mechanism of endocytosis via brain microvascular endothelial cells (BMECs). An experimental in vitro model showed that small EVs under stroke-like conditions are able to bypass the BBB through BMECs, mainly via the transcellular pathway, rather than via tight junctions involved in the paracellular route, to provide their load to the brain parenchyma [112].

Understanding the mechanisms of the passage of MSC-EVs through the BBB represents a prerequisite for the development of EV-based strategy for the treatment of neurodegenerative diseases using, in particular, the intranasal delivery pathway.

### 4.3. Intranasal Route of Delivery

Intranasal (i.n.) administration represents an attractive strategy to deliver pharmacological or cellular substances directly into the brain in a non-invasive way. Several advantages of i.n. delivery are reported [125]: the i.n. route skips the gastrointestinal tract and the hepatic metabolism, increasing absorption and reducing the dose to achieve the beneficial effect [126]. Furthermore, the therapeutic action is rapid and good tolerability is reported.

**Figure 2:**
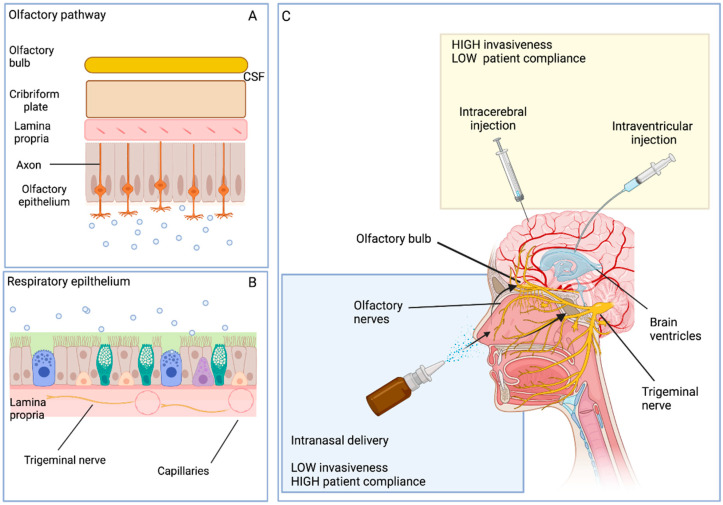
Intranasal delivery of MSC-EVs. MSC-EVs reach the brain through the olfactory bulb directly into the CSF (**A**) or by the trigeminal pathway from the nasal epithelium to the systemic circulation (**B**). The intranasal delivery shows several advantages in terms of invasiveness and patients compliance compared to other direct routes of administration (**C**).

Neurodegenerative diseases are, for the most part, progressive or recurrent and their treatments often require repeated dosages to achieve long-term beneficial effects [127,128]. The possibility to adopt therapeutic regimens of repeated administrations matches with the use of i.n. delivery. Indeed, in terms of patient compliance, repeated i.n. administrations result in improving patient tolerance without extra harm as reported instead for more invasive approaches, which cause embolism, trauma, or infections (Figure 2). The multi-dose therapeutic regimen, with the assistance of devices for auto-administration or simple procedures (drops, aerosol, spray), becomes even more instrumental for patients with neurodegenerative diseases who usually have cognitive and functional issues. Daily i.n. administration of insulin is already reported in AD trials [129]. In addition, other Phase III or IV clinical trials are reported for CNS diseases.

In the context of cellular therapies, beneficial effects of injecting MSCs and their vesicular counterpart via the i.n. route have been reported [130,131]: Losurdo and co-workers used i.n. administration of EVs derived from cytokine-preconditioned MSCs to induce neuroprotection and immunomodulation in 3xTg AD mice, thereby damping the activation of microglial cells, increasing the density of dendritic spines, and modulating the inflammatory status of treated mice [132].

An immunomodulatory response was also observed in EAE mice treated i.n. with EVs from MSCs with an increase in the frequency of Foxp3+ CD25+ regulatory T cells, accompanied by an amelioration of pathological and clinical changes until the end of treatment [133]. Interesting results were also reported on a unilateral 6-OHDA medial forebrain bundle rat model of PD using EVs from human exfoliated deciduous teeth stem cells with a consequent improvement in motor function, which correlated with normalization of tyrosine hydroxylase expression in the striatum and substantia nigra of treated animals [134].

In a recent study, we compared the effect of small EVs isolated from ASCs (ASC-EVs) using two different routes of administration: intravenous (i.v.) and intranasal, in SOD1(G93A) mice, a mouse model of ALS. We demonstrated that repeated i.v. or i.n. administrations of ASC-EVs improved the motor performance in SOD1 mice compared to controls. In addition, the treatment demonstrated a neuroprotective effect on motoneurons in the lumbar section of the spinal cord of treated mice. Therefore, with the same therapeutic effects, the intranasal route turns out to be a minimally invasive, effective, and direct way to convey EVs inside the CNS, bypassing the obstacle of the BBB [85].

The concentration, dose, and volume of administration are important factors that can affect nasal drug delivery to the brain. However, some restrictions or limitations should be taken into consideration, such as the complex anatomy and physiology of the nasal epithelium [135].

The nasal epithelium proves to be permeable to lipidic small nanoparticles, which can cross the epithelium via transcytosis. Furthermore, stem cells and their EVs also cross the barrier in a similar way [133] and the process could be mediated by inflammatory signals that promote trans-epithelial migration [136]. The majority of i.n. delivery studies are conducted on animal models, rodents in particular, and their anatomical structures together with the area of olfactory regions are very different from human ones [137]. Moreover, mucociliary clearance represents a limiting factor in the pharmacokinetics of intranasal drug delivery. The presence of mucociliary clearance may limit the absorption of i.n. injected substances; indeed, one of the important functions of the nasal cavity is the removal of dust, allergens, and bacteria as part of its normal physiological function [138]. In particular, the presence of degrading enzymes such as cytochrome P450, peptidases, and proteases can have an impact on the metabolism of drugs and cellular components [137,139,140]. Several efforts have been made in the field of biotechnology to prolong the residence time of drugs within the nasal cavity by the addition of absorption enhancers, mucoadhesive polymers, in situ gelling agents, enzyme inhibiting agents, and bioactive scaffolds, which result in higher bioavailability [141,142].

Furthermore, the engineering of nanoparticles in general can improve the penetration through the elastic mucin fiber by means of electrostatic changes. Studies by Carlson and co-workers [143] demonstrated that the engineering of the surface of nanoparticles, with a hydrophilic coating of polyethylene glycol (PEG), achieves a significant improvement in their penetration and diffusion across the mucosal barrier.

The possibility to translate the bioengineering applications mentioned above to cellular nanoparticles, and in particular to MSC-EVs, could improve their bioavailability through the i.n. route, resulting in a privileged strategy to treat neurodegenerative diseases.

### 4.4. Functional Modification of MSC-EVs

Advanced studies have attempted to manipulate and modify the surface or the content of stem cell-derived EVs in order to improve their homing capacity and therapeutic potential for specific purposes. The modifications previously described to improve the efficiency of intranasal administration of MSC-EVs represent only partially the bioengineering applications used to improve the therapeutic action of EVs [108,144].

Although intranasal delivery appears to be an advantageous route for the application of MSC-EVs, most preclinical and clinical studies currently report the use of systemic administrations. However, systemic delivery causes high dispersion of EVs throughout the body and a very short residence time in the blood. Improving EV stability in the systemic circulation and protecting their content from enzymatic/proteolytic tissue clearance could be a strategy to increase EV concentration, and enhance the number of vesicles that are able to reach target sites before the clearing action of macrophages [145]. As demonstrated in the tumor model, the binding between the transmembrane protein CD47 and the signal regulatory protein alpha (SIRPα) prevents phagocytosis of EVs by macrophages, thus increasing their blood concentration and improving their delivery to target sites [105,106].

The improvement in the therapeutic action of EVs could be possible through exploiting the targets that are naturally present on EVs (proteins, lipids, or glycans) or through their functionalization with engineered approaches.

In order to prolong the circulation time of EVs, several chemical modifications have been tested, such as PEGylation: the use of a hydrophilic polymer was shown to improve cell specificity of PEGylated EVs and to prolong their circulation times [108,146].

These approaches were also used to target neural cells, avoiding accumulation in no specific sites [123,147]. “Click chemistry” approaches were used to target peptides linked to the EV surface, as shown in a murine model of cerebral ischemia, in order to deliver EVs towards injured areas [107].

An improvement in the therapeutic action of EVs is obtained also by modifying/functionalizing their content: the overexpression of miRNA-21 in EVs from hypoxia-preconditioned MSCs was able to reduce the cognitive deficit and amyloid deposition in AD mice as well as to decrease inflammatory markers [71]. The overexpression of specific miRNA, such as miR-133b, was observed to promote neural plasticity and functional recovery in stroke [109].

Synergistic approaches can potentiate the therapeutic effect of EVs: Peng and coworkers have developed a self-oriented system for intranasal administration of MSC-EVs in a PD mouse model. They exploited MSC-EVs as a nanocarrier cargo overexpressing miR-133 to promote nerve axons growth and recovering neuronal function. Moreover, EVs were also loaded with superparamagnetic iron oxide nanoparticles (SPIONs), in order to orient them across the membrane barriers and release drugs into the cytoplasm of target cells, together with hydrophobic curcumin to alleviate neuroinflammation and clear α-synuclein aggregates [110].

Combining the intrinsic properties of stem cell-derived EVs with a targeted functional modification could prove to be an effective strategy to treat neurodegenerative disorders.

## 5. Translational Applications of MSC-EVs in Patients with Neurodegenerative Diseases

### 5.1. Current Clinical Applications of MSC-EVs in Neurodegenerative Diseases

Preclinical data on EVs-based therapies, as previously discussed, are very encouraging. MSC-EV therapies proved to be much safer and more versatile than cell therapy, despite few clinical results being unavailable still [148,149]. Several studies involving the use of EVs/exosomes are registered on https://beta.clinicaltrials.gov (accessed on 25 January 2023). The majority of those are observational studies focusing on EVs from patient body fluids for diagnostic and prognostic purposes. Promising results have been confirmed in other diseases [150]. Worthy of mention is the clinical trial NCT03384433, which evaluated the stereotaxic injection of MSC-EVs overexpressing miR-124 for the treatment of ischemic stroke and its recurrence. Currently, only two trials including MSC-EVs, and chronic neurodegenerative diseases are registered: depression, anxiety, and dementias (NCT04202770) and Alzheimer’s disease (NCT04388982). In NCT04202770, focused ultrasound was used to enhance the intravenous delivery of EVs from MSCs to the subgenual target for patients with refractory depression, the amygdala for patients with anxiety, and the hippocampus for patients with cognitive impairment. The registered study NCT04388982 evaluates the safety and efficacy of MSC-EVs in patients with mild to moderate dementia, by repeated intranasal administration of MSC-EVs (at low, medium, and high doses) twice a week, respectively, for 12 weeks.

Although there are still few clinical studies currently registered, the interest in these therapies appears to be growing and corroborated by the promising results obtained from preclinical studies (Table 2).

### 5.2. Issues in Clinical Translation of MSC-EVs-Based Therapy

**Figure 3:**
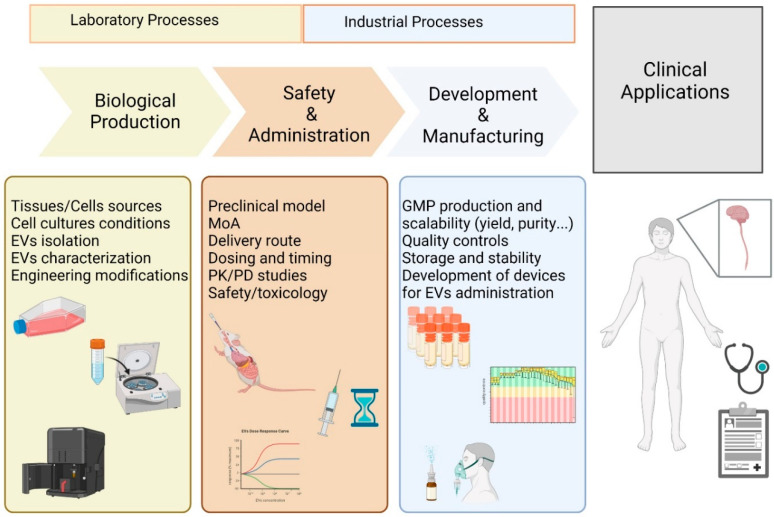
From bench to clinic application of MSC-EVs. Representative overview of the translational development of MSC-EV-based therapies. The use of MSC-EVs in clinical applications requires several key steps: the downstream biological aspects followed by administration and safety studies until the GMP production and regulatory aspects reflect the linear progression from bench to clinic. MoA = mode of action; PK = pharmacokinetic; PD = pharmacodynamic; GMP = good manufacturing practices.

The transition from basic or preclinical research to the clinic still has to overcome several gaps at various levels (Figure 3).

The complex biological nature of MSC-EVs makes it difficult to identify their mode of action, and we can likely expect that the neuroprotective and immunomodulatory effects will not be limited to a single, unique active molecule. The partial identification of the active components of MSC-EVs complicates the definition of the precise mechanism of action of MSC-EVs [155].

On the other hand, many inconsistencies are largely due to the technical difficulties that impact manufacturing processes (identification of the best cellular source, culture and storage conditions, cell type variability, phenotypic instability after cell passaging) [156] as well as non-univocal and standardized methods of extraction and characterization of vesicles. EV detection, isolation, and analysis have been hampered by the limitations of available technologies, and results reported in the last ten years are mostly contaminated by artifacts. To cope with the various problems relating to the standardization of processes, ISEV proposed and updated the Minimal Information for Studies of Extracellular Vesicles (MISEV) guidelines in 2018 to ensure and improve the quality of research on EVs [36].

The transition towards therapeutic application also requires the identification of an effective therapeutic dose to produce a response in humans. However, results are controversial and remain highly variable from study to study. Several parameters, including the previously discussed route of administration, the homing and biodistribution, as well as dosing and timing of the MSC-derived EVs have to be carefully investigated [157].

The majority of preclinical data are obtained in vitro or from rodents such as mice or rats, representing the starting point to identify the minimal effective dose of MSC-EVs for a specific therapeutic application that could be extrapolated for humans. Comparative studies examining the preclinical literature are reported to identify a possible conversion of the dose of EVs from animal treatment to humans [158,159]. However, such comparisons are not sufficient to predict the therapeutic outcome due to their limited translatability to human physiology [160].

Clinical translation would require the use of large animals that could help in pharmacokinetics and formulation studies due to their great similarity to humans. Nevertheless, the use of large experimental models has unaffordable costs, although they would be very suitable, especially for the study of intranasal delivery [161,162].

In addition, the regulatory requirements, associated with good manufacturing practices (GMP) production and scaling for large distribution, should also be clarified and implemented for several aspects such as quality controls or production variability [163].

## 6. Conclusions

In summary, the reported studies represent the proof of concept of the potential of MSC-EVs as a therapeutic opportunity to treat neurodegenerative diseases. The possibility of combining the intrinsic properties of their parental cells with bioengineering modifications could represent a potential improvement for their clinical use. The development of a formulation of EVs that improves their biodistribution and retention in the lesion sites through an appropriate route of administration holds great promise for a facilitated delivery, mainly for CNS diseases. In this regard, the intranasal administration of MSC-EVs represents a flexible treatment, which simplifies the delivery procedure in a direct, efficient way.

Although there are still many challenges to be addressed, the clinical translation of MSC-EVs towards a real therapy represents an interesting frontier for the treatment of neurodegenerative diseases. In recent years, the number of ongoing clinical trials that are actively recruiting patients has been constantly expanding, and the successful translation of EV-based therapeutics in the clinic seems to be more realistic and not so distant, thanks to the rapid advances of nanotechnologies and the support of coordinated studies worldwide.

## Figures and Tables

**Table 1:** Strategies to improve the therapeutic potential of MSC-EVs.

	Methods	Strategies	References
Target identification	Tracking methods	Fluorescent dyes: -NIR labeling -CD63-EGFP -PKH-26	[90] [91] [92]
		-USPIO	[93,94,95,96]
		-GNP	[97,98]
Delivery route	Intranasal administration	Enzymatic breakdown of mucus	[99]
		Chemical methods to alter mucus structure (mucolytics, expectorants, and mucokinetic agents)	[100]
		Addition of absorption enhancers: -Sodiumtauro-24,25-dihydrofusidate (STDHF) -Soybean-derived sterol (SS)	[101,102] [103]
		Protease and peptidase inhibitors	[104]
Bioengineering modifications	Surface modifications	-SIRPα-expressing EVs -CD47-expressing EVs	[105] [106]
	Chemical approaches	-Click chemistry: conjugate functional ligands onto EVs surfaces -PEGylation	[107] [108]
	Content enrichment	-miR-21 overexpression(AD mouse model) -miR-133b overexpression (Stroke rat model) -miR-133 overexpression (PD mouse model)	[71] [109] [110]

NIR = near infrared; USPIO = ultrasmall superparamagnetic iron oxide nanoparticles; GNP = glucose-coated gold nanoparticles; PEG = polyethylene glycol; AD = Alzheimer’s disease; PD = Parkinson’s disease.

**Table 2:** Relevant preclinical studies on MSC-EVs in neurodegenerative experimental models.

Disease	Source of EVs	Route of Administration	Outcomes	References
AD	UMC	i.v.	Reduction in Aβ levels and improvement in cognitive functions	[69]
	BM (hypoxia-preconditioned)	i.v.	Reduction in Aβ levels, anti-inflammatory impact and improvements in learning and memory functions	[71]
	Not reported	i.c.	Promotion of neurogenesis and improvement in cognitive function	[73]
	BM (cytokine-preconditioned)	i.n.	Stimulation of neuroprotection and inhibition of neuroinflammation	[132]
	BM	i.c.	Reduction in Aβ burden and cognitive improvements	[151]
PD	BM	i.v.	Neuroprotection of DA neuron in substantia nigra and upregulation of dopamine levels in striatum	[79]
	DP	i.n.	Improvement in motor functions and normalization of tyrosine hydroxylase expression in the substantia nigra and striatum	[134]
	BM	i.n.	Reduction in α-syn aggregates and functional recovery	[110]
ALS	AT	i.v./i.n.	Neuroprotection of motor neurons, and neuromuscular junctions and improvement in motor performances	[85]
HD	AM (conditioned medium)	i.p.	Amelioration of motor functions	[152]
MS	AT	i.n.	Improvement in motor function, and attenuation of inflammation and demyelination	[133]
	placenta	s.c.	Improvement in motor function and induction of myelin regeneration	[153]
	AT	i.v.	Reduction of demyelination in the spinal cord and immunomodulation	[154]

AD = Alzheimer’s disease; PD = Parkinson’s disease; HD = Huntington’s disease; ALS = amyotrophic lateral sclerosis; MS = multiple sclerosis; UMC = umbilical cord; BM = bone marrow; DP = dental pulp; AT = adipose tissue; AM = amniotic membrane; i.v. = intravenous injection; i.n. = intranasal injection; i.c. = intracerebral injection; i.p. = intraperitoneal injection; s.c. = subcutaneous injection; Aβ = amyloid beta; α-syn = alpha-synuclein.

## Data Availability

Not applicable.
